# A systematic review of emotion regulation in parent-mediated interventions for autism spectrum disorder

**DOI:** 10.3389/fpsyt.2022.846286

**Published:** 2022-09-23

**Authors:** Nicole M. Hendrix, Katherine E. Pickard, Grace E. Binion, Elizabeth Kushner

**Affiliations:** ^1^Department of Pediatrics, Emory University School of Medicine, Atlanta, GA, United States; ^2^Marcus Autism Center, Children's Healthcare of Atlanta, Atlanta, GA, United States

**Keywords:** autism spectrum disorder, emotion regulation, parent-mediated intervention, challenging behavior, systematic review

## Abstract

Autistic individuals are at elevated risk for difficulties with emotion regulation (ER) that emerge early in life and are associated with a range of internalizing and externalizing disorders. Existing interventions that support ER have focused on school-age autistic children and adolescents as well as adults. Proactive approaches to improving ER in early childhood are thus needed, as is understanding the approaches by which ER skills can be feasibly supported in this young population. This review summarizes how ER has been measured within parent-mediated interventions for children at or under the age of 6 years and the extent to which ER is measured concurrently with or distinctly from observable behaviors that have been referenced in existing literature as externalizing or challenging behavior. Using PsycInfo, EBSCOhost, and PubMed databases, we searched for peer-reviewed journal articles published through August 2021, that focused on the use of parent-mediated interventions targeting ER and/or challenging behavior. The systematic search resulted in 4,738 publications; following multi-stage screening, the search yielded 20 studies. Eighteen of 20 studies were designed to target challenging behavior using manualized curricula or behavior analytic methodologies and assessed child outcomes through validated caregiver rating scales and/or direct behavioral observation. One study measured changes in ER as secondary to the social communication skills that were targeted in the intervention. Only one study specifically supported ER skill development and measured changes in ER as the primary intervention outcome. Findings highlight the need for better assessment of ER outcomes within the context of parent-mediated interventions for toddlers and young autistic children.

Autism spectrum disorder (ASD) is a neurodevelopmental disorder that emerges in early childhood characterized by differences with social communication and restricted, repetitive interests and behavior (1). In addition to these core symptoms, autistic individuals [see commentary on identity-first language in Bottema-Beutel et al. (2)] experience mental health disorders, including anxiety and depression, at higher rates than clinically referred individuals without ASD (3, 4). Current and lifetime prevalence rates for anxiety in autistic individuals are estimated at 27% and 42%, respectively, with current and lifetime prevalence rates for depression estimated at 23% and 37% (5, 6). Co-occurring mental health conditions can interfere significantly with autistic individuals' quality of life—including community, academic, and vocational participation—and increase feelings of loneliness and social isolation (7–9).

One mechanism which may explain elevated mental health challenges in autistic individuals is emotion regulation [ER, (7, 10–13)]. Emotion regulation encompasses processes that an individual uses to modulate or change their emotional state to achieve a goal (14, 15). ER appears to serve as a transdiagnostic mental health risk factor within both non-autistic and autistic populations [e.g., (16, 17)] with specific brain regions consistently implicated across populations, albeit with emerging evidence for variable patterns of activation [e.g., (18)]. These emotion-related, self-regulatory processes develop within the first few years of life and are supported by both extrinsic (i.e., a caregiver soothing a child) and intrinsic factors (i.e., an individual modulating their internal emotional responses). In early childhood, emotional dysregulation has often been conceptualized within the context of irritability [see review in (19)], and as manifesting in varied ways including as the presence of externalizing or challenging behavior. In this review, we use the term ER to indicate the emotion-related, self-regulation processes that are intrinsic and voluntary and that are used to modulate or alter emotional experiences irrespective of the amount or type of emotion being experienced (15, 20).

Autistic individuals are at elevated risk for difficulties with ER (4, 6, 21, 22). Though ER difficulties are not encompassed within ASD diagnostic criteria, they have been shown to be associated with core autistic features (17, 23, 24). These ER difficulties are evident across the lifespan (7, 17, 25, 26) and are associated with internalizing and externalizing disorders in late childhood, adolescence, and adulthood (10). For both autistic and non-autistic individuals, ER abilities are related to temperamental characteristics, including effortful control and executive functioning (27). These temperamental characteristics emerge early in childhood and are often areas of difficulty for autistic individuals (12, 28).

Most research examining the development of ER in autistic individuals has examined these processes in adolescents and adults, although a growing body of research has evaluated the development and use of ER in children. This research has shown that school-age autistic children experience greater emotion dysregulation than their non-autistic peers and use more fragmented ER strategies in response to frustrating situations (25, 29, 30). Although research is somewhat limited in very young children and toddlers, a recent systematic review found that ER challenges are also more evident in autistic toddlers and preschool-age children when compared to young children without ASD (12). These early ER difficulties have been shown to be relatively stable and predictive of internalizing and externalizing symptoms, as well as social and behavioral functioning even at a young age (17, 23).

## Treatments supporting autistic individuals' ER

Given the high prevalence of ER difficulties in autistic individuals and their association with mental health challenges, many treatments have been developed to support ER in autistic individuals (31–33). Examples include those that specifically target ER as well as those that support ER skills as part of broader mental health and social skills interventions (22). Interventions designed to support ER skills often aim to increase emotion awareness, distress tolerance, and ER strategy use. Some interventions take a mindfulness-based approach to supporting ER (34, 35), whereas broader mental health and social skills treatments typically include sessions devoted to teaching emotion identification and management strategies alongside other intervention elements (22). In addition to treatments that teach ER skills to support internalizing symptoms, a handful of other studies have targeted the acquisition of ER skills to prevent or reduce child behaviors like hitting others and refusing parental instructions [e.g., (36, 37)].

A growing body of research supports the efficacy of interventions supporting ER in autistic individuals, yet this research has almost exclusively occurred in older autistic youth and adults (34, 38). Moreover, research aimed at supporting ER in younger, school-age children (i.e., ages 5 to 7 years) often uses traditional skills-based programs (e.g., affective education in conjunction with teaching stress management strategies) that have been developed for older youth and adapted for younger populations (39). These studies have had promising results, with participating children showing improvements in ER skills (39). As difficulties with ER emerge early in autistic children (40, 41), more proactive and developmentally appropriate approaches to supporting ER skills are needed during critical periods in which ER is developing.

## Parent-mediated interventions as a context for early ER treatment

One means to proactively support the development of effective ER skills in preschool-age autistic children is through parent-mediated interventions. Parent-mediated interventions are a treatment model in which a therapist systematically teaches or coaches a caregiver to implement therapeutic strategies with their child within meaningful home and community routines (42). A growing evidence base suggests parent-mediated approaches are efficacious in reducing challenging behaviors (43) and in improving social communication in young autistic children (44). Parent-mediated interventions may also support family outcomes, including parent-child interactions and caregiver empowerment (45).

Parent-mediated intervention may be particularly well-suited to support ER skills in young children with ASD given the instrumental role that caregivers play in the development of ER for both autistic and non-autistic children (46, 47). For example, caregivers support ER development through reciprocal and transactional interactions, modeling of ER strategies, and scaffolding the use of ER strategies (48). Research has demonstrated that caregivers use a variety of strategies to support their autistic children in regulating their emotions, many of which resemble the strategies used by caregivers of non-autistic children but that are also responsive to their child's developmental needs (46). Although caregivers support ER development in both autistic and non-autistic children, little is known about the extent to which caregivers have been leveraged to bolster ER skill development through participation in parent-mediated interventions.

## ER difficulties and externalizing or challenging behaviors

When considering the extent to which parent-mediated interventions support the development of ER skills, it is also important to include parent-mediated interventions focused on externalized, challenging behavior, as these behaviors may ultimately represent internal ER processes. For example, in autistic individuals, the experience of heightened anxiety (i.e., an internal emotional state) is associated with increased reporting of aggressive behaviors [e.g., (9, 49)]. It is also possible that anxiety (i.e., and internal state rooted in ER processes) may contribute to observable challenging behaviors over time [e.g., a child who engages in aggressive behaviors that result in escape or avoidance of anxiety-provoking contexts (50)]. The co-occurrence between ER processes and external behaviors suggests the need to consider that challenging behaviors may at times represent external cues of dysregulated emotional states or behavioral attempts at regulating emotional states (51).

The inclusion of parent-mediated interventions focused on externalized, challenging behaviors is also important given the historical emphasis of autism early intervention on the reduction of challenging behaviors or the replacement of challenging behaviors with other skills (43). Although a large body of research has examined the impact of early ASD treatment on challenging behavior, it is unclear the extent to which the ER processes underlying these behaviors are also measured as an outcome alongside challenging behavior [e.g., (52)]. The current study aims to address this gap by providing a systematic review of ER and/or challenging behavior as an outcome within parent-mediated interventions. A more inclusive criteria was chosen to capture the overlap between the ER difficulties and challenging behavior, as well as the prevalence of autism intervention literature targeting challenging behavior. Measuring challenging behaviors was made given the overlap between challenging behaviors and ER difficulties [e.g., (21, 50)]. Study aims were to: 1) evaluate the frequency with which ER is assessed in the context of parent-mediated interventions within early childhood, 2) determine the extent to which ER is measured concurrently with or distinctly from challenging behavior; and 3) examine whether the reviewed parent-mediated interventions aim to directly support ER skills.

## Methods

### Search strategy

In line with standard practice, the Preferred Reporting Items for Systematic Reviews and Meta Analyses [PRISMA (53)] were used to guide this review. Database selection and search string were developed for comparability with recent published systematic reviews of ER in ASD (12, 17). Search strings from these studies were modified to ensure capture of parent-mediated interventions for a broad range of emotional and behavioral challenges which might be related to ER. We concurrently searched PsycINFO, EBSCOhost, and PubMed to identify empirical studies of parent-mediated interventions for young autistic children published through August 2021. The following search strings were used to search study titles and abstracts in each of these databases: (autism OR Asperger OR pervasive developmental disorder) AND (emotion regulation OR emotional regulation OR emotion management OR affect regulation OR emotional competence OR effortful control OR challenging behavior OR problem behavior OR aggression OR self-injurious behavior OR non-compliance OR destruction) AND (intervention OR therapy OR treatment OR psychotherapy OR parent-mediated). Both titles and abstracts were searched to ensure all articles which reported on relevant constructs were identified, even when search constructs were not the primary focus of a study or treatment.

### Eligibility criteria

Inclusion and exclusion criteria were established prior to search completion. Studies were included if they met the following criteria: (1) included individuals who had a diagnosis of ASD (including diagnoses of autistic disorder, Asperger's syndrome, and/or pervasive developmental disorder—not otherwise specified); (2) included participants with a mean age at or below 6 years or 72 months; (3) included an intervention in which the therapists or researchers worked directly with caregivers and systematically used coaching strategies like role play and/or practice with feedback to support the caregiver in learning and implementing strategies to support child outcomes [i.e., parent-mediated intervention, (42)]; and (4) included an outcome measure of children's ER or challenging behavior. Studies were excluded if they met the following criteria: (1) were reviews, expert opinion commentaries, theory papers, or individual case studies; (2) examined developmental or neurodevelopmental disorders broadly without distinguishing between diagnostic groups; (3) were not available in English; (4) were not published (e.g., unpublished dissertations, conference abstracts); or (5) did not target ER and/or challenging behavior as a primary or secondary treatment outcome. Although the exclusion of unpublished literature may bias results such that they cannot be said to reflect the totality of research in this domain, such exclusion is in line with the goal of the current review—to assess the state of the published literature.

### Screening and data extraction

**Figure 1:**
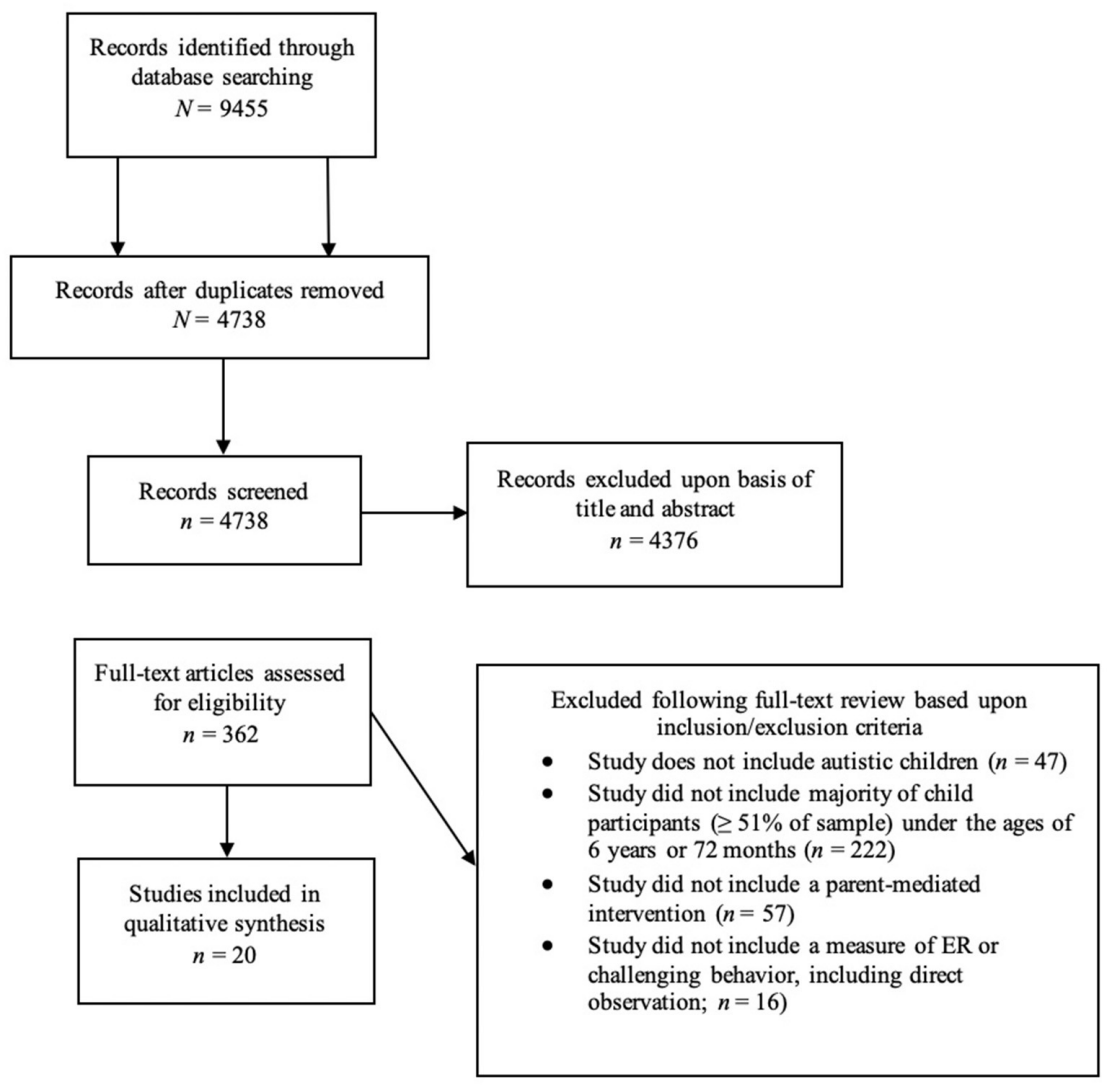
Flowchart of included and excluded studies.

An online platform for data storage, screening, and extraction in systematic reviews (DistillerSR; Evidence Partners, Ottawa, Canada) was used in this review. The initial search yielded 9,455 records, of which 4,716 were identified as duplicates. Duplicate removal resulted in 4,738 records; these records then underwent multi-stage screening. First, titles and abstracts were screened by one of three coders for initial eligibility. Of these, 1,648 were excluded due to absence of a parent-mediated intervention as either the focus of treatment or a component of treatment delineated in terms of systematic teaching and/or coaching strategies; 1,602 were excluded due to not being an original empirical study; 1,058 were excluded for not including autistic children; 62 were excluded for not including a measure of ER or challenging behavior; and 6 were excluded for one or more of the previous reasons. Full-text copies of the remaining 362 articles which either met inclusion criteria or for which eligibility could not be determined were retrieved and screened by this same team of coders. At the full text screening stage, all articles were screened by two coders. Inter-rater reliability for full-text screening was calculated using Cohen's Kappa. Kappas for individual screening items ranged from 0.74 to 0.85, indicating excellent agreement on all items (weighted overall *k* = 0.81). Any discrepancies which impacted inclusion/exclusion decisions (*n* = 2) were resolved in discussion with the first author (N.H.). At the full text screening stage, an additional 342 articles were excluded due to not meeting inclusion criteria. This resulted in a final sample of 20 articles (see Figure 1).

A data extraction form was created to extract information related to study populations; study aims; and treatment targets, methods, and outcomes. Descriptive information was extracted to characterize variability within the published literature. This information included year of publication and country in which the study was conducted. In accordance with the aims of this review, the following information was extracted from all articles: intervention name or focus, treatment setting, treatment duration, sample mean age, sample size, putative treatment target, presence or absence of a measure purporting to assess ER, method of ER measurement (if present), presence or absence of a measure purporting to assess challenging behavior, and method of challenging behavior measurement (if present).

## Results

The present search resulted in 20 peer-reviewed parent-mediated intervention studies for ER and/or challenging behavior (see Table 1). Fifteen studies took place in the United States (U.S.); the remaining five studies took place in Canada (54), China (55), Iran (56), Italy (57), and the Netherlands (58). Of the full sample, 10 studies took place primarily in participants' homes, and 10 took place within clinical centers or university settings. One study took place in a clinical setting that provided early intensive behavioral intervention services in addition to participant homes (57). Six studies were conducted using telehealth; three of these studies took place primarily in participants' homes (59–61), while three other studies were conducted with parent-child dyads attending telehealth sessions at a clinical site near their homes (62–64).

**Table 1:** Summary of studies addressing emotion regulation (ER) and/or challenging behavior (CB) via parent-mediated intervention (PMI).

**Study**	**Country**	**Diagnosis**	**Intervention name (focus)**	**Primary Setting**	**Session number and length**	**Child *M* age in months (range)**	**PMI *n***	**ER measured**	**Challenging behavior measured**
Bearss et al. (65)	U.S.	ASD	Research Units in Behavioral Intervention (Behavior)	Clinic	11–13; 60- to 90-min	57.6	89	No	Yes
Bearss et al. (62)	U.S.	ASD	Research Units in Behavioral Intervention (Behavior)	Telehealth	11–13; 60- to 90-min	69.6	14	No	Yes
Duifhuis et al. (58)	Netherlands	ASD	Pivotal Response Treatment (Behavior)	Clinic	20; 45-min	68.4 (49–100)	11	No	Yes
Gerow et al. (59)	U.S.	ASD	Functional communication training (Behavior)	University or Telehealth	Not described	51 (36–60)	4	No	Yes
Gulsrud et al. (46)	U.S.	ASD	Joint attention (Social Communication)	Clinic	24; 60-min	30.6 (21–36)	34	Yes	No
Huynen et al. (66)	U.S.	DD	Planned Activities Training (Behavior)	Home	5; 90-min	51 (24–72)	4	No	Yes
Leung et al. (55)	China	DD	Happy Parenting Program (Behavior)	University	8; 120-min	48.7	62	No	Yes
Lin and Koegel (67)	U.S.	ASD	Self-management (Behavior)	Home	20–24; 60-min	65.3 (57–80)	3	No	Yes
Lindgren et al. (63)	U.S.	ASD/DD	Functional communication training (Behavior)	Telehealth	≥25; 60-min	50 (21–84)	107	No	Yes
Lindgren et al. (60)	U.S.	ASD	Functional communication training (Behavior)	Telehealth	12; 60-min	49.7 (29–83)	21	No	Yes
Moes and Frea (68)	U.S.	ASD	Functional communication training (Behavior)	Home	Not described	41.3 (39–43)	3	No	Yes
Rispoli et al. (69)	U.S.	ASD	Regulation of Emotional Lability in Autism Spectrum Disorder through Caregiver Supports (ER)	Home	8; 60- to 120-min	53.8 (37–79)	5	Yes	Yes
Rivard et al. (54)	Canada	ASD	Prevent Teach Reinforce for Young Children (Behavior)	Home	12; 60-min	48	23	No	Yes
Robertson et al. (70)	U.S.	ASD	(Behavior)	Home	≥10; 60-min	50 (34–66)	2	No	Yes
Ruppel et al. (71)	U.S.	ASD	Balance Program (Behavior)	Home	Not described	48.5 (39–55)	4	No	Yes
Suess et al. (64)	U.S.	ASD	Functional communication training (Behavior)	Telehealth	3; 15-min	59.2 (29–85)	5	No	Yes
Shiri et al. (56)	Iran	ASD	Family-Based Management of Behavioral Excesses of Autism Program (Behavior)	Home	10; 90-min	35.3 (24–47)	17	No	Yes
Strauss et al. (57)	Italy	ASD	Early intensive behavioral intervention (Behavior)	Clinic, Home	Not described	55.7 (26–81)	24	No	Yes
Tsami and Lerman (61)	U.S.	ASD	Functional communication training (Behavior)	Telehealth	Not described	60 (36–72)	5	No	Yes
Zlomke and Jeter (72)	U.S.	ASD/DD	Parent-Child Interaction Therapy (Behavior)	Clinic	16; 60- to 90-min	51.5 (24–96)	14	No	Yes

Empirically validated interventions were represented within the pool of reviewed studies, in addition to more novel, pilot intervention programs [e.g., (55, 69)]. The empirically validated interventions included those targeting challenging behaviors such as aggression and property destruction [e.g., Parent-Child Interaction Therapy (PCIT); Functional Communication Training (FCT)], as well as those intended to target social communication and adaptive behaviors. Variability was observed with respect to intervention duration and sample size as well. Although over half of the studies included intervention session lengths of 60- to 120-min, treatment sessions for some studies were as short as 15-min in duration (64). When considering studies that explicitly reported the number of treatment sessions, studies ranged from three to greater than 25 sessions. With respect to study methodology and sample size, 11 studies employed group designs to examine differences in participant behavior from pre- to post-treatment (range: 11–107 participants), and nine studies relied upon visual analysis using single case research designs (range: 2–5 participants).

Two studies (62, 65) used a manualized parent training intervention for challenging behavior with similar structure, intervention duration, and content within the targeted age group. A total of 180 participants participated in the Bearss et al. (65) multi-site trial, with 89 children randomly assigned to the parent training condition. In Bearss et al.'s (62) study, an additional 14 participants completed this manualized intervention with an emphasis instead on intervention feasibility when delivered via telehealth.

Eighteen of 20 studies included stated treatment targets related to challenging behaviors like self-injury, property destruction, and aggression. A total of six studies used FCT (59–61, 63, 64, 68). Of note, one study (63) included a sample that partially overlapped with two other studies (60, 64). A seventh study explicitly integrated FCT within a broader behavior analytic package for behaviors like whining, yelling, aggression, and throwing items (71). Five studies employing behavior analytic methodologies to decrease the rate or frequency of child challenging behaviors also targeted language or social communication (57, 60, 61, 70, 71).

Only two studies assessed ER in young autistic children as part of intervention (46, 69). Gulsrud et al. (46) examined ER as a secondary construct within an existing intervention dataset targeting joint attention within 34 parent-child dyads. Child ER was assessed across the intervention through behavioral coding of child negative affect as well as categorized child self-regulation strategies. In contrast, Rispoli et al. (69) developed the Regulation of Emotional Lability in Autism Spectrum Disorder through Caregiver Supports (RELACS) parent-mediated intervention, which primarily promoted ER skills in autistic preschool-age children. Reductions in child emotion dysregulation in four of five participants using a caregiver report measure at completion of the intervention were reported (69).

## Discussion

Goals of this systematic review were to examine the extent to which ER or challenging behavior is measured within the context of parent-mediated interventions for young autistic children, the extent to which ER is measured alongside or distinct from challenging behavior, and the extent to which ER is specifically targeted within these interventions. Overlap between ER and externalized challenging behaviors [e.g., (9, 51)] guided the decision for inclusion criteria in this review to be intentionally broad to capture studies that assessed either or both constructs. Specifically, this review included any empirical study that evaluated a parent-mediated intervention for autistic children under the age of 6 years and measured either ER or challenging behavior as an outcome using a validated scale or through behavioral observation.

A total of 20 studies met these criteria. Almost every study that met the inclusion criteria targeted challenging behavior using manualized curricula focused on behavior management principles, targeted behavior analytic interventions, or broader behavior analytic treatment packages. Studies targeting challenging behaviors, such as aggression toward others, self-injury, property destruction, and disruptive vocalizations like screaming, measured challenging behavior as a primary outcome through use of caregiver rating scales and behavioral data collection. Only one study included an intervention specifically targeting ER skill development (69). Finally, one additional study measured changes in a child's ER over the course of their participation within a parent-mediated intervention aimed at supporting joint attention skills (46). Overall, the included interventions were conducted both in-person and via telehealth, in-home and clinic-based, and ranged from three to greater than 25 sessions. In addition to the inclusion of large-scale trials that examined manualized parent-mediated interventions for challenging behavior [e.g., (65)], a strength of this review is that it captured nine single case study designs that centered on direct observation of behavior change. Many of these studies used parent-mediated intervention rooted in behavioral principles to support the use of functional communication and behavioral flexibility [e.g., (64, 67)].

To date, systematic reviews and meta-analyses of parent-mediated interventions have examined those that support social communication development as well as interventions that focus on reduction of challenging behaviors (43, 44). The extent to which parent-mediated interventions could also be used to support the development of ER skills remained largely unexamined. This limitation was important to address as high rates of ER difficulties in autistic individuals begin as early as toddlerhood (12, 41) and predict the development of interfering mental health challenges including anxiety and depression in childhood, adolescence, and adulthood (17, 23). Although a number of treatments have been designed to facilitate ER skill development, many of these are designed to support individuals in middle childhood through adulthood—well after the emergence of ER difficulties (22). The essential role of caregivers in ER development for both autistic and non-autistic children (48) as well as the potential for proactively mitigating the impact of ER difficulties over time suggests that parent-mediated interventions in early childhood may represent a natural opportunity to support ER and to examine ER as an intervention outcome.

It was not unexpected that nearly all reviewed interventions emphasized challenging behavior—particularly in the context of operant learning paradigms—when considering the historic emphasis of the field on interventions using behavior analytic principles, the presence of autism insurance mandates that have increased access to behavior analytic treatment and the relatively high co-occurrence of behaviors such as self-injury and aggression in autistic children (73–76). Of the studies targeting challenging behavior, none measured ER as a secondary outcome. This finding indicates that literature on treatments of challenging behavior in young autistic children is not measuring or supporting ER as a core process in parallel to externalizing behaviors that may contribute to these behaviors. The lack of ER measurement within the existing literature was surprising given research suggesting that some challenging behaviors may be rooted in internal ER processes [e.g., (49)].

Of the reviewed studies, only one examined a parent-mediated intervention that explicitly supported ER skills in young autistic children (69). This intervention supported caregivers in modeling ER strategies, using relevant visual supports, and managing other challenging behaviors. Within the multiple-probe single case design, four out of five children demonstrated parent-reported reductions in emotion dysregulation from pre- to post-treatment (69). Although ER was not a core target of the joint attention intervention in Gulsrud et al. (46), this study found that children decreased their expression of negative emotion over the course of treatment, while mothers increased their use of emotional scaffolding (46). Their results may suggest that parent-mediated interventions targeting social communication are able to support ER skill development through supporting caregivers' use of affect to increase social engagement and maintain regulation (77). In doing so, these interventions may support co-regulation, even when not an explicit intervention target.

The limited measurement of ER within the reviewed studies may, in part, be due to difficulty measuring internal ER processes as distinct from more observable behaviors such as aggression or self-injury. Measurement may also be hindered by few ER rating scales that have been validated in autistic toddlers or toddler with other developmental delays (17). Behavioral coding schemes developed to assess observable ER processes in young children used in conjunction with rating scales may strengthen the validity of ER measurement. In future work, behavioral coding schemes may be used to assess ER within this age group (17) and may complement observational coding that is already utilized within parent-mediated intervention [e.g., (46)]. Work is also actively being done to extend indirect ER measures with strong psychometric properties [e.g., (78)] to toddler- and preschool-age autistic populations, creating other avenues for illuminating ER difficulties in young autistic children.

Limitations of this review include the narrow age range considered as studies were included only if they had a sample of children with a mean age of 6 years or younger. This age range was chosen given the early emergence of ER challenges in autistic children and the intention of the review to examine interventions that may support ER skill development prior to the onset of more consequential ER or mental health difficulties. In doing so, this age range excluded parent-mediated interventions targeting challenging behavior in slightly older youth [e.g., (79)]. Moreover, we did not include interventions targeting ER development by teaching skills directly to young autistic children. Studies to date have adapted individual, skill-based programs for young children ages 5–7 years with promising findings (39). The selection of parent-mediated intervention was made in response to evidence demonstrating the role of caregivers in supporting ER development through co-regulation (47, 48), making parent-mediated intervention an appropriate context for supporting ER development. Finally, it is possible that the search string used [e.g., “autism” as opposed to “autis^*^”] may have limited the sensitivity of the search. However, the current search strategy was used to align this review with those previously conducted on similar topics (12, 17).

Future directions include improved measurement of ER within the context of parent-mediated interventions. It will be critical to measure ER alongside challenging behavior to help tease apart whether changes in ER skills co-occur with changes in behavior or serve as the foundation by which children replace challenging behaviors with more adaptive skills. Although caregiver rating scales of ER in young autistic children are limited, measurement of ER could include behavioral observation of negative affect and emotional reactivity (17). Consideration of ER could also be given within interventions targeting a wider constellation of social communication skills. Examination of ER alongside social communication outcomes will be particularly important with the overlap in ER and core autistic features (24) and findings that parent scaffolding may change within intervention targeting social communication. Relatedly, it will be important to measure caregiver behaviors that are thought to be related to ER development to better understand whether changes in caregiver responsivity, emotional scaffolding, and/or stress relate to changes in child ER skills. Finally, although there is not yet consensus on factors that moderate outcomes within parent-mediated interventions (80), it may be important to consider ER as a treatment moderator. Its relationship to challenging behaviors and the impact of these types of behaviors on treatment engagement and participation imply relationships to be explored in future studies.

Understanding how evidence-based interventions can proactively support ER skills in young autistic children is integral to enhancing research and practice. There is a growing body of evidence to support the positive impact of parent-mediated interventions on child and family outcomes. Thus, the application of these intervention models in the context of ER may help to build ER skills in very young children while offsetting mental health difficulties. Further, the intertwined relationship between ER, challenging behavior, and social communication provides an opportunity to explore how ER skills may change in response to parent-mediated interventions, the caregiver behaviors that may support these changes (e.g., co-regulation), and the extent to which changes in ER are related to social communication and behavioral outcomes.

## Data availability statement

Publicly available datasets were analyzed in this study.

## Author contributions

NH: coordinated the systematic review, screened and reviewed abstracts and full texts, wrote the results, and edited manuscript. KP: wrote the introduction and discussion, screened and reviewed abstracts and full texts, and edited manuscript. GB: wrote the methods and screened abstracts. EK: conducted reliability for screened abstracts, supported revisions, and supported formatting and references. All authors contributed to the article and approved the submitted version.

## Conflict of interest

The authors declare that the research was conducted in the absence of any commercial or financial relationships that could be construed as a potential conflict of interest.

## Publisher's note

All claims expressed in this article are solely those of the authors and do not necessarily represent those of their affiliated organizations, or those of the publisher, the editors and the reviewers. Any product that may be evaluated in this article, or claim that may be made by its manufacturer, is not guaranteed or endorsed by the publisher.
